# Evaluation of the cost effectiveness of exenatide versus insulin glargine in patients with sub-optimally controlled Type 2 diabetes in the United Kingdom

**DOI:** 10.1186/1475-2840-7-24

**Published:** 2008-08-11

**Authors:** Anette Woehl, Mark Evans, Anthony P Tetlow, Philip McEwan

**Affiliations:** 1 School of Mathematics, Cardiff University, Cardiff, UK; 2 Department of Diabetes, Endocrinology and Metabolism, University Hospital of Wales, Heath Park, Cardiff, UK; 3 Cardiff Research Consortium, Health Park, Cardiff, UK

## Abstract

**Objective:**

Exenatide belongs to a new therapeutic class in the treatment of diabetes (incretin mimetics), allowing glucose-dependent glycaemic control in Type 2 diabetes. Randomised controlled trial data suggest that exenatide is as effective as insulin glargine at reducing HbA_1c _in combination therapy with metformin and sulphonylureas; with reduced weight but higher incidence of adverse gastrointestinal events. The objective of this study is to evaluate the cost effectiveness of exenatide versus insulin glargine using RCT data and a previously published model of Type 2 diabetes disease progression that is based on the United Kingdom Prospective Diabetes Study; the perspective of the health-payer of the United Kingdom National Health Service.

**Methods:**

The study used a discrete event simulation model designed to forecast the costs and health outcome of a cohort of 1,000 subjects aged over 40 years with sub-optimally-controlled Type 2 diabetes, following initiation of either exenatide, or insulin glargine, in addition to oral hypoglycaemic agents. Sensitivity analysis for a higher treatment discontinuation rate in exenatide patients was applied to the cohort in three different scenarios; (1) either ignored or (2) exenatide-failures excluded or (3) exenatide-failures switched to insulin glargine. Analyses were undertaken to evaluate the price sensitivity of exenatide in terms of relative cost effectiveness. Baseline cohort profiles and effectiveness data were taken from a published randomised controlled trial.

**Results:**

The relative cost-effectiveness of exenatide and insulin glargine was tested under a variety of conditions, in which insulin glargine was dominant in all cases. Using the most conservative of assumptions, the cost-effectiveness ratio of exenatide vs. insulin glargine at the current UK NHS price was -£29,149/QALY (insulin glargine dominant) and thus exenatide is not cost-effective when compared with insulin glargine, at the current UK NHS price.

**Conclusion:**

This study evaluated the relative cost effectiveness of insulin glargine versus exenatide in the management of Type 2 diabetes using a published model. Given no significant difference in glycaemic control and applying the additional effectiveness of exenatide over insulin glargine, with respect to weight loss, and using the current UK NHS prices, insulin glargine was found to be dominant over exenatide in all modelled scenarios. With current clinical evidence, exenatide does not appear to represent a cost-effective treatment option for patients with Type 2 diabetes when compared to insulin glargine.

## Background

The progression of Type 2 diabetes is driven by progressive *β*-cell dysfunction and increased insulin resistance, which results in hypoglycaemia due to difficulty of achieving glycaemic control. Typically, lifestyle modifications such as diet and exercise fail to achieve and give way to the administration of oral hypoglycaemic agents (OHAs) in order to maintain glucose control. In addition to tolerability issues for patients, the inability of OHAs to stem the decline in *β*-cell function  [1] commonly lead to the introduction of exogenous basal insulin to maintain normoglycaemia [2]. Traditionally regarded as a drastic measure in Type 2 diabetes, physicians are increasingly favouring earlier introduction of basal insulin to control hyperglycaemia and minimise the associated micro- and macrovascular complications of diabetes [3,4]. Whilst undoubtedly clinically effective, use of insulin regimens also carries some problems, namely:

• an inability to control mealtime glucose excursion [5],

• increased risk of severe hypoglycaemia [6,7],

• the need for complicated dose-titration [8], and

• weight gain [9].

Hypoglycaemia of any severity has a profound effect on patients' quality of life [7] and is regarded as the single greatest obstacle to achieving normoglycaemia [10]. In addition to reduced quality of life, hypoglycaemia results in substantial direct medical cost and lost productivity [11].

Insulin glargine (Lantus™) is an analogue of human insulin with a prolonged duration of action and once-daily dosing. In Type 2 diabetes, the principal emergent benefit is significantly reduced risk of all forms of hypoglycaemia over Neutral Protamine Hagedorn (NPH) [12]. However, initiation of insulin glargine still requires careful dose titration to an appropriate level over a period of time. This is essential for successful treatment of diabetes and the avoidance of hypoglycaemia [13]. However, recent trial evidence has suggested that insulin glargine could be introduced earlier to achieve glycaemic goals [14] and a further study showed that adding insulin glargine to OHA therapy had a positive effect on treatment satisfaction and quality of life (QoL) without complaints related to hypoglycaemia [15]. Insulin glargine is currently not recommended by the National Institute for Health and Clinical Excellence (NICE) for routine use for people with Type 2 diabetes, but can be considered for people with Type 2 diabetes who require assistance from a third party to administer their insulin injections, who have recurrent symptomatic hypoglycaemic episodes or who would otherwise need twice-daily basal insulin injections in combination with oral antidiabetic drugs. Using their own model (based on United Kingdom Prospective Diabetes Study (UKPDS) 68 [16]), NICE concluded that human insulin analogues are the most cost-effective option and glargine was estimated to be cost-effective in Type 2 diabetes patients at increased risk of hypoglycaemia [17]; the NICE guidelines are due to be reviewed and republished in early 2009.

The incretin mimetics are emerging as a significant new class of hypoglycaemic agents that regulate glucose homeostasis similarly to endogenous glucagon-like peptide-1 (GLP-1). Exenatide (Byetta™), the first such compound to be licensed by the European Medicine Agency (EMEA) [18], enhances glucose-dependent insulin secretion, regulates glucagon release, and delays gastric emptying thereby reducing hyperglycaemia in a similar manner to GLP-1. Unlike the short-lived GLP-1, exenatide resists metabolism by dipeptidyl peptidase-IV giving it a pharmacokinetic profile suitable for chronic administration [19]. Exenatide is recommended for use in the treatment of Type 2 diabetes in combination with metformin, and/or sulphonylureas in patients who have not achieved adequate glycaemic control on maximally tolerated doses of these oral therapies and is given twice daily by subcutaneous injection. Exenatide was approved by the Scottish Medicines Consortium (SMC) in June 2007, as an adjunct therapy to patients with Type 2 diabetes who, despite currently taking metformin and/or a sulphonylurea either singly or in combination, fail to achieve adequate glycaemic control [20]. Exenatide, however, is not currently recommended by NICE for routine use for people with Type 2 diabetes [17] and should only be considered for people with Type 2 diabetes, who have a body mass index (BMI) over 35 kg/m^2^, specific problems arising from high weight, inadequate blood glucose control (glycosylated haemoglobin (HbA_1c_) >7.5 %), prescribed conventional oral therapy and where other high cost therapies would otherwise be commenced. Using their own model (based on UKPDS 68 [16]), NICE estimated that exenatide was not cost-effective in any scenario [17] and, furthermore, exenatide has been linked with occurrences of acute pancreatitis [21].

The only published head-to-head randomised controlled trial (RCT) [22] of exenatide versus glargine, reported similar improvements in the overall glycaemic control in patients with Type 2 diabetes sub-optimally controlled with combination OHAs at maximal doses. For secondary endpoints, patients treated with exenatide experienced significantly fewer nocturnal hypoglycaemic events (0.9 vs. 2.4 events per patient per year; a 63% reduction), but higher incidence of daytime hypoglycaemia (6.6 vs. 3.9 events per patient per year; a 69% increase) and achieved significant weight loss from baseline (Δ -2.3 kg), whereas insulin glargine-treated subjects gained weight of a similar magnitude (Δ +1.8 kg) by the study endpoint. The principal drawback reported in exenatide treated patients was nausea (in 57.1% of subjects) and vomiting (in 17.4%), which led to withdrawal of almost 10% of exenatide-treated patients from the study protocol. This finding is consistent with evidence from other published phase 3 placebo-controlled trials [23]. By comparison, less than 1% of those exposed to insulin glargine withdrew due to adverse events. The most common adverse event reported with insulin glargine was nasopharyngitis (in 9.0%). Within this trial setting, however, the dosage of glargine was very low with an average of 25 international units (IU)/day. This does not reflect clinical practise, where the dosage is typically 42 IU/KG [24]. A higher dose with glargine will lead to better glycaemic control, but also more weight gain and will have an effect on hypoglycaemic events [25,26]. Regarding patient reported outcomes, Boye and colleagues published the patient reported outcomes measured from the same head-to-head trial [22] and showed no significant difference in improvement of health utility or quality of life [27].

The increasing global financial burden of chronic health conditions is fuelling a rising demand for value for money when introducing new health technologies. In line with many other countries, in the United Kingdom (UK), NICE currently accepts as cost-effective, "those interventions with an incremental cost-effectiveness ratio of less than £20,000 per QALY (quality adjusted life year) and that there should be increasingly strong reasons for accepting as cost effective interventions with an incremental cost-effectiveness ratio of over £30,000 per QALY [28]." A recently published study of Ray et al. [29] showed exenatide to have a cost-effectiveness ratio of £22,420 vs. insulin glargine; however, a major limitation of this study was that it used an estimated UK cost of exenatide based on the US price. The study reported exenatide as dominant (less costly and more effective) when marketed at 20% of the US price in the UK. The study of Ray et al. used cohort details from the Heine trial [22] that were then applied to a previously published cost effectiveness model [30], the CORE Model.

The objective of this analysis was to prospectively evaluate the cost-effectiveness of exenatide from a UK NHS perspective, using insulin glargine as a comparator and the current UK NHS price for exenatide and also applying the cohort profiles and published data from the Heine trial [22]. The model, which has been previously published [31], used to evaluate the cost-effectiveness was a discrete event simulation (DES) model for patients with Type 2 diabetes using UKPDS-derived risk functions [16] and a multivariate regression model for the utility associated with hypoglycaemia [7].

## Methods

### Modelling approach and model

This evaluation was undertaken within the context of the UK National Health Service (NHS), and used the NHS as its perspective (payer's perspective). The method used was a cost-utility analysis (CUA) intended to determine the cost per quality adjusted life years gained (QALYs gained) comparing exenatide to insulin glargine as adjunct therapy to maximal doses of metformin and sulphonylurea in Type 2 diabetes.

The modelling approach used was a discrete event simulation (DES) model of people with Type 2 diabetes using UKPDS-derived risk functions for development of vascular complications [16] and a multivariate regression model for the utility decrement associated with hypoglycaemia [7]. The details of the model and its validation have been published [32], but a brief description is given below.

The model simulates a cohort of 1000 subjects over a 40-year time horizon. The specific events modelled were ischaemic heart disease (IHD), myocardial infarction (MI), congestive heart failure (CHD), stroke, blindness in one eye, end stage renal disease (ESRD) and amputation, in addition to diabetes-related and all-cause mortality; furthermore, the model additionally predicts severe, nocturnal, and symptomatic hypoglycaemic events. The results presented represent an average of one hundred first order Monte Carlo simulations. The baseline characteristics of the subjects from Heine trial [22] (Table 1) were applied and are generally consistent with the profile of insulin-treated patients with Type 2 diabetes in the UK, as older and obese (Body Mass Index (BMI) greater than 30 kg/m^2^).

**Table 1:** Baseline patient characteristics.

Variable	Type 2 diabetes
Age (years)^†^	59
Gender (% male)^†^	56%
BMI (kg/m^2^)^†^	31.9
Weight (kg)^†^	89.8
Height (metres)^†^	1.67
Ethnicity (% black)^†^	1%
Total cholesterol (mmol/L)^‡^	5.2
HDL cholesterol (mmol/L)^‡^	1.04
Systolic blood pressure (mmHg)^‡^	136
HbA_1c _(%)^†^	7.1
Peripheral vascular disease (%)^‡^	30%
Smoking (%)^‡^	0
Risk of severe hypoglycaemia*	0.462
Number of nocturnal hypos (year)^†^	2.4
Number of symptomatic hypos (year)^†^	6.8

^†^Data from Heine et al[22], ^‡ ^data from UKPDS baseline cohort[16], * data from Leese et al[6]

The model first simulates treatment of the cohort with basal insulin glargine plus OHAs. At the beginning of each time period, checks were made for specific fatal or non-fatal events. The order in which these events occurred was randomised. If a fatal event occurred, all costs, life years and quality adjusted life years were accumulated and the simulation ended for that individual. The simulation then selects the next individual and the process begins again. Assuming a subject does not die in any specific year then following the 'check for events' stage, a simulated subject's disease state is updated and any appropriate decrement in health utility was then applied together with associated costs. The simulation time clock is then advanced and if the end of the simulation time horizon has been reached, the simulation ended for that individual and the process starts again with the next individual. Once all individuals had been simulated, the process ends and all summary statistics are calculated for that particular run of the model.

The second run of the model for treatment with exenatide plus OHAs utilised exactly the same patient cohort data as the first run but applied the alternative treatment effects and costs (Table 2). After applying any differential effects to the patient data, the model was then re-initialised and run through in exactly the same manner as for the first run.

**Table 2:** Treatment and comparator costs applied in the model.

**Description**	**Cost**	**Source**
*Insulin glargine group*		
price per 1000 IUs	£26.00	BNF No. 55[33]
Glucose test strip	£0.302	2006 DoH Prescription Cost Analysis[34]
Lancet	£0.055	2006 DoH Prescription Cost Analysis[34]
*Exenatide group*		
10 ug BD, per 28 days	£68.24	BNF No. 55[35]
*Both groups*		
Metformin per day (500 mg QDS)	£0.21	BNF No. 55[33]
Gliclazide per day (320 mg per day)	£0.25	BNF No. 55[33]

### Effectiveness

Three significant endpoints emerged from the Heine trial [22] which were included in the model as treatment effects. Overall, patients in the exenatide group had a higher rate of hypoglycaemic events then patients in the insulin glargine group (7.3 events/patient-year vs. 6.3 events/patient-year). Nocturnal hypoglycaemia was 63% lower in the exenatide group than the insulin glargine group (0.9 events/patient-year vs. 2.4 events/patient-year), but patients in the exenatide group had a 69% higher incidence of daytime hypoglycaemia than patients the insulin glargine group (6.6 events/patient-year vs. 3.9 events/patient-year). No differences in severe hypoglycaemia were recorded and therefore the model was run using previously published rates [6].

At the 26-week trial endpoint average patient weight was 4.1 kg lower in the exenatide group compared to the insulin glargine group. For each group, change in weight (kg) over time (weeks) had begun to plateau by the study endpoint, a pattern echoed by the long-term extension trials of exenatide [23]. Thus, this difference was conservatively applied as the maximum weight improvement for exenatide patients. Differential levels of HbA_1c _were not modelled as the trial showed no significant difference in glycaemic control between insulin glargine and exenatide.

Finally, discontinuation rates due to adverse events (Δ 8.9% of intention-to-treat (ITT) patients) were modelled using three approaches: either completely ignored; exenatide-discontinuations removed from analysis; and exenatide discontinuations switched to insulin glargine.

### Estimates of financial costs

The financial costs applied to the simulation model are detailed in Table 3, and summarised as follows. Costs of medical treatment were obtained from UK sources and were indexed to year 2007 with UK Treasury rates [32].

**Table 3:** Event, Maintenance and therapy costs (indexed to £UK 2007)

**Variable**	**Cost**	**Source**
Ischaemic Heart Disease, fatal event	£0	UKPDS Study No.65†
Ischaemic Heart Disease, Non-fatal event	£2,388	UKPDS Study No.65†
Ischaemic Heart Disease, Maintenance	£601	UKPDS Study No.65†
Myocardial Infarction, Fatal event	£1,404	UKPDS Study No 65†
Myocardial Infarction, Non-fatal event	£4,961	UKPDS Study No.65†
Myocardial Infarction, Maintenance	£566	UKPDS Study No.65†
Congestive Heart Failure, Fatal event	£0	UKPDS Study No.65†
Congestive Heart Failure, Non fatal event	£2,707	UKPDS Study No.65†
Congestive Heart Failure, Maintenance	£769	UKPDS Study No.65†
Stroke, Fatal event	£4,124	UKPDS Study No.65†
Stroke, Non-fatal event	£2,885	UKPDS Study No.65†
Stroke, Maintenance	£304	UKPDS Study No.65†
Blindness, Event	0	O'Brien et al. [37]
Blindness, Maintenance	£1,013	O'Brien et al. [37]
Dialysis, Event and Maintenance	£20,049	Baboolal et al. [38]
Amputation, Fatal event	£10,311	UKPDS Study No.65†
Amputation, Non-fatal event	£10,311	UKPDS Study No.65†
Amputation, Maintenance	£366	UKPDS Study No.65†
Hypoglycaemia, Event	£93.85	Leese GP et al. [6]; DCCT hypoglycaemia report*

† Data from: Clarke P, Gray A, Legood R, Briggs A, Holman R: **The impact of diabetes-related complications on healthcare costs: results from the United Kingdom Prospective Diabetes Study (UKPDS Study No. 65). ***Diabetic Medicine *2003, **20**:442–450.

* Data from: The DCCT Research Group: **Epidemiology of severe hypoglycemia in the diabetes control and complications trial**. *American Journal of Medicine *1991, **90**:450–459.

**Treatment and comparator **Insulin glargine cost was calculated on a per kilogram basis, costed at the current UK weighted average wholesale prices for Lantus across the different preparations [33] to reflect the UK market. A 0.28 IU/kg/day dose regimen was assumed for insulin glargine, based on the average daily requirement reported at the study endpoint [22]. In common with the Heine trial protocol [22], where daily glucose monitoring was not required for patients assigned to receive exenatide, the additional cost of one reagent tests strip and one lancet per day was applied. In each case, the weighted average cost was derived from the 2006 Department of Health (DOH) Prescription Cost Analysis [34]. As neither blood glucose testing meters nor finger-pricking devices are available on the NHS, they did not represent a cost to the health payer and were excluded from the analysis. The exenatide treatment cost was estimated using the UK market launch price [35]. 5 microgram twice daily (BD) was assumed for initiation for a month and after this a 10 microgram BD maintenance dose, this follows the NHS recommendations [36]. In both the insulin glargine and exenatide simulated cohorts the cost of metformin and gliclazide (representing 1^st ^line sulphonyureas) administered were modelled at their respective maximum daily dosages [33]. The model assumes these first line therapies are well tolerated to the maximum dose and patient management will typically involve titrating to the maximum daily dose and then subsequent therapy will be added to this and titrated appropriately.

**Macrovascular event costs **Macrovascular event costs are split into either fatal or non-fatal costs and were applied in the year in which the event occurred. Maintenance costs for those subjects surviving were applied in all subsequent years until either the end of the simulation time horizon or until the subject died.

**Blindness (in one eye) **Subjects were assumed to incur blindness in one eye only. The initial cost related to the event was assumed to be equal to zero, with subsequent maintenance costs applied annually thereafter from published data [37].

**Nephropathy **Dialysis costs were annual weighted mean costs for peritoneal dialysis (£15,534; 71%) and haemodialysis (£33,516; 29%) [38].

**Peripheral vascular disease **was modelled as the occurrence of an amputation and had a single cost associated with the event and a subsequent annual maintenance cost.

**Severe hypoglycaemia **costs are applied to severe hypoglycaemia events only and are based on the costs of hospitalised treatment reported in Tayside, UK. The Diabetes Control and Complications Trial (DCCT), estimated that 20% of all severe hypoglycaemic episodes result in a hospital intervention, either emergency room attendance or inpatient hospitalization. The most comprehensive data relating to treatment modality of severe hypoglycaemic episodes is reported by Leese et al. [6], from their observational study of registered diabetics in Tayside, Scotland, UK. In this study, 34% of severe hypoglycaemia episodes were managed by an ambulance attendance, 46% by accident & emergency (A&E) consultation, and the remaining 20% were hospitalized as inpatients, representing 5% of all estimated severe hypoglycaemia (hospitalized or not) for this population. Over the 12-month study period, Leese et al report 244 episodes totalling £92,078 of healthcare resources, some £377.37 per episode. Given this represents only a fifth of all severe episodes [39], the average known cost across all episodes in 1998 (year of Leese' data collection) is estimated to be £75.47. Indexed to 2007, the average cost of treating all DCCT-defined severe hypoglycaemia is £93.85.

### Health-related utility

Utility estimates were taken from either the UKPDS study [16], or generated via the Health Outcomes Data Repository (HODaR) database [40,41]. The utility decrements associated with each specified event have been specified previously [31] and are shown in Table 4. The model assumes that multiple diabetes related complications have an additive effect on utility; that is, the combined utility decrement for a person experiencing both an MI and stroke event would be the sum of the two individual utility values. The same utility decrement was applied in years subsequent to the year in which the event occurred. Utility associated with hypoglycaemia events is handled somewhat differently. Statistical models were developed that related the frequency and severity of hypoglycaemia to fear of hypoglycaemia, and subsequently to changes in health-related utility [7]. The relationship between hypoglycaemia and utility was modelled using a two stage approach in which equations were derived describing the relationship between hypoglycaemia and the fear of hypoglycaemia (measured via the Hypoglycaemia Fear Score (HFS)). Subsequently the relationship between changes in HFS was related to health utility, measured using the EQ5D [42]. The equations showed that the marginal effects of the occurrence of at least one severe hypoglycaemic episode was associated with a 5.881 change in HFS score and that unit changes in the natural log of the number of symptomatic events and the square root of the number of nocturnal events was associated with a 1.773 and 1.054 change in the HFS score respectively. Each unit increase in HFS was associated with a utility of decrement of 0.008 (for severe and symptomatic) and 0.007 for nocturnal hypoglycaemia. The disutility associated with the fear of hypoglycaemia is applied throughout each simulated year if a hypoglycaemic event occurs; if simulated patients do not experience an event then no disutility is applied. Hypoglycaemia is the only adverse event accommodated in the model; the analysis does not model complications such as acute pancreatitis and therefore is somewhat conservative with respect to glargine.

**Table 4:** Clinical endpoints modelled and utility decrements applied

**Health state**	**Utility**	**Source**
Ischaemic Heart Disease	-0.09	UKPDS Study No.68[16]
Myocardial Infarction	-0.055	UKPDS Study No.68[16]
Congestive Heart Failure	-0.108	UKPDS Study No.68[16]
Stroke	-0.164	UKPDS Study No.68[16]
Blindness in one eye	-0.074	UKPDS Study No.68[16]
ESRD	-0.305	Lee et al. [42]
Amputation	-0.28	UKPDS Study No.68[16]

Important differences exist between exenatide and glargine that may also affect quality of life; in particular, changes in weight and a different side effect profile. A number of studies have shown exenatide to be associated with weight reduction [43,44] whilst insulin is associated with weight gain [45,46]. Furthermore, exenatide has been shown to have more frequent gastrointestinal side effects. A number of studies have reported changes in utility associated with changes in weight (or more specifically, BMI). Importantly, these studies are cross-sectional in design and do not capture changes in utility associated with an individuals' experience of their own weight loss/gain and, therefore, the application of such utility gains may overstate the effects of weight change. This view is corroborated by results from a randomised trial comparing patient reported outcomes in 549 subjects randomised to either insulin glargine or exenatide. Despite expected differences in weight change, side effect profile and dosing frequency, insulin glargine patients exhibited a non-significant increase in EQ5D compared patients receiving exenatide (0.03 versus 0.02 utility gain, p = 0.35) [47].

The results presented here did not accommodate any utility changes associated with gastrointestinal side effects or weight change. Furthermore, consistent with longitudinal studies assessing health utility and insulin initiation, no utility decrement was applied to therapy type [48].

### Other economic analysis details

All prices are all adjusted to 2007 values (UK£) using the UK Treasury gross domestic product (GDP) deflator formula [32]. Where necessary, costs and benefits were discounted at 3.5% per year in accordance with current NICE guidance [28].

## Results

### Number of end points forecast in each arm

The model was designed to output the frequency (per 1,000 patients) in each of a range of pertinent vascular events and mortality that are listed in Table 5 for the three main scenarios.

**Table 5:** The forecast frequency of vascular endpoints and cost-effectiveness using insulin glargine or exenatide per 1,000 patients over 40 years under the three discontinuation scenarios.

	**No discontinuation**			**Failures excluded**			**Failures switched**		
			
	**Exenatide**	**Glargine**	**Δ**	**Exenatide**	**Glargine**	**Δ**	**Exenatide**	**Glargine**	**Δ**
**Macrovascular**									
Ischaemic Heart Disease	122.2	121.2	*-1.06*	111.1	121.4	*10.32*	121.8	121.4	*-0.36*
Myocardial Infarction	387.4	388.2	*0.82*	351.9	387.7	*35.79*	386.1	387.7	*1.61*
Congestive heart Failure	84.9	92.6	*7.68*	77.7	92.3	*14.59*	85.4	92.3	*6.87*
Stroke	98.9	99.7	*0.74*	90.1	98.3	*8.19*	99.2	98.3	*-0.89*
									
**Microvascular**									
Retinopathy	55.5	55.4	*-0.03*	50.7	55.4	*4.73*	55.3	55.4	*0.11*
Nephropathy	14.1	13.8	*-0.29*	12.7	14.1	*1.41*	14.1	14.1	*-0.01*
Neuropathy	12.9	12.9	*0.04*	11.8	12.9	*1.13*	13.1	12.9	*-0.14*
									
**Hypoglycaemia events**									
Nocturnal	12901.2	34259.8	*21358.6*	11754.3	34269.6	*22515.3*	14804.9	34269.6	*19464.7*
Symptomatic	94479.7	55672.2	*-38807.5*	86080.9	55688.1	*-30392.8*	91090.4	55688.1	*-35402.3*
Severe	6622.0	6597.5	*-24.5*	6036.2	6598.1	*561.9*	6626.2	6598.1	*-28.2*
									
**Fatal**									
Macrovascular	453.5	458.2	*4.68*	412.8	457.3	*44.53*	453.3	457.282	*4.008*
Microvascular	12.7	12.7	*-0.02*	11.6	12.8	*1.18*	12.9	12.752	*-0.154*
Other	532.8	528.1	*-4.68*	486.0	529.0	*42.99*	532.8	529.0	*-3.845*
									
**Cost effectiveness**									
Discounted costs	£14,567,526	£9,280,312		£13,255,912	£9,296,371		£14,092,624	£9,296,371	
Discounted QALYS	7,683	7,864		7,000	7,865		7,703	7,865	
			
**ICER (£/QALY)**	**Dominant (-£29,149)**			**Dominant (-£4,579)**			**Dominant (-£29,657)**		

Note: the model takes an average of a series of runs in order for the resultant values to become stable. In taking this average non-integer values can be generated.

### Ignoring discontinuation rates

In the first scenario where no exenatide treatment discontinuations was considered, there was an overall difference of 21,359 nocturnal episodes of hypoglycaemia, in favour of exenatide, but 38,808 more daytime episodes of hypoglycaemia for patients treated with exenatide. Overall 24.5 severe hypoglycaemic events were predicted for patients treated with exenatide. The biggest difference in vascular endpoints occurred for congestive heart failure (CHF), where use of exenatide results in 7.7 fewer events on average; this difference being driven by the improved BMI profile associated with exenatide.

### Removing patients discontinuing from the analysis

With exenatide treatment discontinuations excluded from the analysis, the number of clinical endpoints predicted are fewer in the exenatide group (with the exception of daytime hypoglycaemic events); however, this observation is due to fewer patients available in the model to experience clinical endpoints.

### Switching patients discontinuing exenatide to insulin glargine

Switching exenatide discontinuations to insulin glargine produces similar results to ignoring discontinuation rates altogether. The number of nocturnal hypoglycaemic events with exenatide reduces to 19,465 and the number of additional daytime hypoglycaemic events increases by 35,402. The number of expected CHF events avoided with exenatide is 6.87 when compared with insulin glargine. Under these conditions, mortality due to macrovascular events is still favourable to exenatide with 4.0 deaths avoided, but this scenario is the least positive result to exenatide compared with 4.7 macrovascular deaths saved in the baseline scenario and 43.0 macrovascular deaths saved when exenatide treatment failures are excluded.

### Total costs and QALYS forecast in each arm

Applying the current UK price of exenatide, total treatment costs over the simulation period associated with exenatide use were significantly higher than for insulin glargine. Ignoring discontinuations rates resulted in the discounted total costs (per 1,000 patients) of £14,567,526 and £9,280,312 for exenatide and insulin glargine, respectively (Δ ≈ £5.3 M, Table 5). The total and difference in quality adjusted life years (QALYs) is also listed in Table 5; with 160 fewer QALYs associated with exenatide. Although there were fewer nocturnal hypoglycaemic events associated with exenatide, their contribution to improving quality of life was offset by higher daytime events and overall higher severe hypoglycaemic events when compared to insulin glargine. Using the current multivariate hypoglycaemia model each nocturnal hypoglycaemic event results in a 0.68% decrement in utility, whereas one severe hypoglycaemic event leads to a 4.40% utility decrement [7].

### Incremental cost effectiveness ratios

Under the no discontinuation scenario, the mean incremental cost effectiveness ratio (ICER) was-£29,149 per QALY gained, with insulin glargine being less costly and more effective than exenatide. Under the most conservative scenario of excluding exenatide failures, the dominance of insulin glargine persisted with a mean ICER of -£4,579 per QALY gained. The scenario where exenatide failures were switched to insulin glargine produced similar results with insulin glargine remaining cheaper and more effective than exenatide (mean ICER of -£29,657 per QALY gained).

### Sensitivity analysis

As a sensitivity analysis, the input factors for the model were varied to reflect long term outcomes and clinical practice for glargine and exenatide. Open label extension of exenatide trials showed that the glycaemic control in the long term was similar to the effect shown in the Heine trial. However weight reduction was continuing up to a maximum of 5.3 kg over 3 years. There was no evidence of the rate of hypoglycaemic events in published the open label extensions [49-51]. Glargine studies applying a more clinically relevant dosage scenario – on average a dose of 53 IU/day – showed that glycaemic control could be more improved than shown in the Heine trial; on average a further reduction of 0.65% in HbA_1c_. On the other hand weight increased on average by 3.0 kg and nocturnal hypoglycaemic events increased on average to 3.55 events/patient-year and symptomatic events on average to 5.65 events/patient-year. Using the same hypoglycaemic rates for exenatide as reported in the Heine trial, the nocturnal hypoglycaemia rate would be 75% lower in the exenatide group than the insulin glargine group, and incidence of daytime hypoglycaemia would be 17% higher in the exenatide group than in the insulin glargine group. Applying this evidence of glycaemic control, weight control and dosage to the model showed that exenatide in comparison to glargine was still estimated to be more costly and a less effective option with a cost-effectiveness ratio of £6,365 (glargine dominant). Using the evidence of increased hypoglycaemic events when using a higher dosage of glargine the results were similar and exenatide in comparison to glargine was still estimated to be more costly and a less effective option with a cost-effectiveness ratio of £6,884 (glargine dominant). The complete results of the two sensitivity scenarios are shown in Table 6.

**Table 6:** Sensitivity analysis for the forecast of frequency of vascular endpoints and cost-effectiveness using insulin glargine or exenatide per 1,000 patients over 40 years (no discontinuation scenario).

	**Long term scenario** ** (without hypoglycaemia risk change)**			**Long term scenario** ** (with hypoglycaemia risk change)**		
		
	**Exenatide**	**Glargine**	**Δ**	**Exenatide**	**Glargine**	**Δ**
**Macrovascular**						
Ischaemic Heart Disease	122.1	115.2	-6.95	122.1	115.2	-6.95
Myocardial Infarction	387.2	367.1	-20.12	387.2	367.1	-20.12
Congestive heart Failure	78.2	87.2	9.07	78.2	87.2	9.07
Stroke	97.5	95.7	-1.86	97.5	95.7	-1.86
						
**Microvascular**						
Retinopathy	55.8	49.2	-6.59	55.8	49.2	-6.59
Nephropathy	14.0	14.0	0.01	14.0	14.0	0.01
Neuropathy	13.1	10.1	-3.04	13.1	10.1	-3.04
						
**Hypoglycaemia** ** events**						
Nocturnal	12,749	35,113	22,364	12,742	51,939	39,197
Symptomatic	94,627	57,059	-37,568	94,907	82,663	-12,244
Severe	6,626.5	6,760.3	133.8	6,626.5	6,760.3	133.8
						
**Fatal**						
Macrovascular	449	428.4	-20.5	449	428.4	-20.5
Microvascular	13.1	11.2	-1.91	13.1	11.2	-1.91
Other	536.8	559.4	22.59	536.8	559.4	22.59
						
**Cost effectiveness**						
Discounted costs	£14,552,192	£12,505,945		£14,552,192	£12,505,945	
Discounted QALYS	7,688	8,009		7,687	7,984	
		
**ICER (£/QALY)**	**Dominant (-£6,365)**			**Dominant (-£6,884)**		

## Discussion

For this analysis, a previously published UK diabetes Type 2 model was used to evaluate the introduction of exenatide in patients with sub-optimally controlled Type 2 diabetes to the UK NHS. As a comparator, insulin glargine, a long-acting once daily human insulin analogue, was chosen. The results of the study reflect the long-term projection of the findings of the Heine trial [22]. Using the baseline profiles and treatment effects outlined here the economic evaluation conducted found that insulin glargine dominated exenatide.

A recently published study of Ray et al. [29] evaluating the cost-effectiveness of exenatide vs. insulin glargine using the same input from the Heine trial [22] showed different results to our findings. Ray et al. demonstrated that exenatide has a cost-effectiveness ratio of £22,420 vs. insulin glargine at an estimated 100% US price. Furthermore, it was estimated that exenatide is dominant (cost and QALY saving), when marketed at 20% of the US price in the UK. A cost-effectiveness ratio below the recommended £20,000/QALY [28] was achieved at 80% of the US price, which approximately equals the current UK market price. Using the same Heine trial data [22] as utilised in this study, Ray et al. [29] estimated that exenatide is a cost-effective treatment option in patients with sub-optimally controlled Type 2 diabetes in the UK. Ray et al. [29] modelled their baseline scenario (100% US price) over a time horizon of 35 years and reported higher direct medical costs for exenatide and insulin glargine £29,401 and £19,489 per patient respectively) and higher QALYs (14.62 and 14.51 per patient respectively) than we derived in our analyses for exenatide and insulin glargine (cost: (£14,568 and £9,280 per patient respectively; QALYs: 7.68 and 7.86 per patient respectively) using a time horizon of 40 years. Both studies showed that lifetime medical costs are higher for patients with exenatide and insulin glargine and Ray et al.'s [29] estimate was much higher over a shorter time period than ours.

The main difference between the outcomes reported by Ray et al. [29] and our own study were the QALY estimates with Ray et al. [29] estimating a QALY gain for patients treated with exenatide, while our analysis showed a QALY gain for patients treated with insulin glargine. Reviewing the input factors for the model of Ray et al. [29] showed that the authors used more evidence from the Heine trial [22] in favour of exenatide than we used in our study. While there is clear evidence for a difference in hypoglycaemic events, weight loss and patients with nausea, there was no clear evidence of any significant difference in other factors favouring exenatide. Ray et al. [29] included lower systolic blood pressure (SBP), lower total cholesterol (TC), lower low-density lipoprotein (LDL) and lower triglycerides in patients treated with exenatide. These risk factors are influential predictors of future diabetes-related complications and improvements reported in the Heine trial [22] were non-significant. We did not see this as evidence for a significant treatment effect and therefore did not include these in our model. As outlined in the methods section, we did not include disutility associated with weight gain, gastrointestinal side effects or insulin initiation. The justification for this approach being the analysis of patient reported outcomes in a head to head comparison of exenatide and glargine. Furthermore, while cross sectional studies have demonstrated a disutility of insulin use compared to oral agents [52], prospective longitudinal studies have shown that insulin therapy does not impact on quality of life [53].

The major drawback of the Heine trial [22] was that the dosing of glargine did not reflect clinical practice. Within the trial design, the dosing of glargine was very low, with an average of 25 IU/day. A higher dose of glargine naturally leads to improved glycaemic control, but also more weight gain and, potentially, hypoglycaemic events. Our sensitivity analyses showed that applying long term data to the exenatide arm and a more clinically relevant dosing scenario for glargine produced results consistent with the base case findings.

As with all modelling simulations, our economic evaluation has a number of strengths and weaknesses. The model can only be as reliable as the predictive algorithms and other data that were used in its construction. The model was based on contemporary data largely drawn from the same source – the United Kingdom Prospective Diabetes Study; however, the dependence of our findings on the UKPDS model is not without drawbacks. In particular, the UKPDS outcomes model does not completely capture the complexities of all diabetes related complications. For example, complications such as peripheral neuropathy, ulceration, gross proteinuria or proliferative retinopathy and macular oedema are handled crudely, primarily due to insufficient patient numbers experiencing these events in the UKPDS study. This is largely due to the inclusion criteria of the UKPDS study in which patients were newly diagnosed with Type 2 diabetes with no pre-existing cardiovascular complications. Type 2 diabetes, however, is primarily characterised by macrovascular complications and the equations published from this study represent a robust approach to describing the relationship between the major complications occurring in Type 2 diabetes and modifiable risk factors. No modelled economic evaluation can ever provide point estimates that are demonstrably precise; nevertheless, the fact that exenatide is significantly more costly than insulin glargine, but provides only marginal benefits with respect to BMI provides some degree of confidence that the conclusions presented here are reliable.

This study strictly applied those statistically significant differences between exenatide and insulin glargine emergent reported by Heine et al [22]. Although not necessarily an accurate prediction of the performance of exenatide in day-to-day practice, it is, nevertheless, consistent with NICE economic modelling guidance [28], and therefore appropriate. The study did not include utility estimates regarding weight changes and adverse events due to treatment. Published utility data related to weight changes are not well reported, but preliminary analyses of HODaR data [40], including longitudinal weight and utility data suggested that utility changes start to become significant if a patient gains/loses a great amount of weight (>5 kg) and it is dependent on the initial weight (the higher the BMI the more likely the patient benefit from weight loss). Certain other factors not reported by the Heine trial [22] could also have had an impact on these outcomes. For example, what was the utility decrement associated with nausea and vomiting in the exenatide group? Did the significant weight loss among exenatide-treated subjects improve utility, in spite of gastro-intestinal symptoms, and was the weight loss a result of nausea, pancreatic and other gastro oesophageal problems? How did lipid profiles change as a result of weight loss experienced in the exenatide arm? At the time of writing, these data are not available in the public domain but, in their absence, it is reasonable to conservatively assume there were no significant differences between the groups otherwise they would presumably have been reported in the trial-related publication.

## Conclusion

This study could have a number of implications for policy makers. Given therapeutic equivalence of exenatide and insulin glargine concerning glycaemic control it is likely that a forthcoming NICE appraisal of exenatide possibly would apply the lower £20,000 threshold. This economic evaluation showed that glargine, when used in the treatment of Type 2 diabetes patients, was dominant in comparison to exenatide. Maintaining the current UK price difference between insulin glargine and exenatide, the recommendation of exenatide should be considered cautiously as a cost-effective alternative to insulin glargine for tertiary treatment of Type 2 diabetes given the evidence present for exenatide.

## Competing interests

The authors declare that they have no competing interests.

## Authors' contributions

PMc conceived the study. AW and TT prepared the model. All authors contributed to the design of the protocol. AW drafted the manuscript. All authors have read and approved the final manuscript.
